# Synthesis and Properties of Degradable Poly(3-hydroxybutyrate-*co*-3-hydroxyvalerate) [P(3HB-*co*-3HV)] Derived from Waste Fish Oil

**DOI:** 10.3390/polym17162171

**Published:** 2025-08-08

**Authors:** Tatiana G. Volova, Evgeniy G. Kiselev, Alexey G. Sukovatyi, Natalia O. Zhila, Kristina Yu. Sapozhnikova, Natalia D. Ipatova, Peter O. Shishatskii

**Affiliations:** 1 Institute of Biophysics SB RAS, Federal Research Center “Krasnoyarsk Science Center SB RAS”, 50/50 Akademgorodok, Krasnoyarsk 660036, Russia; volova45@mail.ru (T.G.V.); evgeniygek@gmail.com (E.G.K.); a.sukovatiy@yandex.ru (A.G.S.); kristina.sap@list.ru (K.Y.S.); ipatovahatal@gmail.com (N.D.I.); shishatskaya@inbox.ru (P.O.S.); 2 Basic Department of Biotechnology, School of Fundamental Biology and Biotechnology, Siberian Federal University, 79 Svobodnyi Av., Krasnoyarsk 660041, Russia

**Keywords:** waste fish oil, biodegradable polyhydroxyalkanoates, renewable materials, copolymers, biosynthesis

## Abstract

The article presents the results of the first successful synthesis of degradable microbial copolymers of 3-hydroxybutyrate and 3-hydroxyvalerate [P(3HB-*co*-3HV)] by the wild-type strain *C. necator* B-10646 using waste fish oil (WFO) obtained from the heads of *Sprattus sprattus balticus*. Samples of copolymers with 3HV monomer contents from 11.9 to 59.7 mol.% were synthesized with fractional and controlled feeding of potassium valerate, a precursor of 3HV monomers, into the bacterial culture. Samples synthesized on WFO with different contents of 3HV monomers had a reduced degree of crystallinity (36.5% and below), and close average molecular weight (390–573 kDa), with polydispersity of 2.6–3.0, and retained thermal stability, with a gap between the melting point and the thermal degradation temperature of over 100 °C. The thermal behavior of the samples, including the kinetics of exothermic crystallization and spherulite formation, was studied. Demonstrating the possibility of using WFO for the effective synthesis of P(3HB-*co*-3HV) with macroinclusions of 3HV monomers without deterioration of their properties is important for expanding the raw material base, reducing costs and increasing the availability of these promising bioplastics.

## 1. Introduction

The development of new polymeric materials is a key task in greening the technosphere and developing high technologies. The relevance of these studies lies in the need to develop industrial processes that are compatible with the environment, as well as the growing demand for environmentally friendly materials that are integrated into the biosphere’s cycles. These materials are in high demand across various areas of human activity. Polyhydroxyalkanoates (PHAs)—degradable polymers of microbiological origin—contribute to the currently implemented strategy of gradually replacing non-degradable synthetic plastics derived from oil with new-generation materials. These “green” plastics are promising for use in various fields including biomedicine and pharmacology, agriculture and public utilities, the textile and packaging industries, the automotive industry, construction, etc. [1,2,3,4,5,6,7,8,9,10].

The history of the discovery of these promising biopolymers is associated with Beijerinck, who in 1888 discovered granules in bacterial cells during staining [11]. Subsequently, these granules were identified and described as intracellular inclusions of the polymer *β*-hydroxybutyric acid, also known as poly(3-hydroxybutyrate) [P(3HB)] [12,13]. The global oil crisis that began in 1973 and the subsequent rise in oil prices prompted OPEC member countries, which control the plastics market, to recognize the need to search for alternative methods of producing polymeric materials. In 1976, in the UK, the ICI was the first to implement a commercial process for the microbiological synthesis of P(3HB) using sugar-containing substrates [14]. Significant interest was aroused by the report that this polymer is thermoplastic, similar to polypropylene [15]. The identified biodegradability and biocompatibility of P(3HB) [16] increased interest in the bacterial process for producing this material and stimulated the number of publications.

Poly(3-hydroxybutyrate) is currently the most studied representative of the PHA family that is produced on an industrial scale. However, the high degree of crystallinity characteristic of P(3HB), the proximity of the melting point and thermal degradation, and the low rate of crystallization complicate the processing of this polymer, resulting in products with low mechanical strength. Over time, it becomes brittle because of the formation of large spherulites and the occurrence of secondary crystallization processes [17,18,19].

The impetus for expanding research on PHAs was the discovery that microorganisms can synthesize multicomponent polymers. A polymer with properties different from P(3HB) was isolated from activated sludge. Chromatographic analysis of the sample showed that in addition to the dominant monomers 3-hydroxybutyrate, it contained 3-hydroxyvalerate, 3-hydroxyhexanoate, and 3-hydroxyoctanoate [20]. This was the first copolymeric PHA discovered. To date, over 150 different polymers from the PHA family have been described. In the seminal work of Professor A. Steinbüchel [21], it was proposed that the diversity of PHAs should be divided into three groups according to the length of the C-chain: short-chain length (SCL), which consists of acids with a carbon chain length of 3 to 5 carbon atoms; medium-chain length (MCL), which includes acids with 6 to 14 carbon atoms; and long-chain length (LCL), which contains acids C17 and C18. It was quickly discovered that PHAs with different chemical compositions have different physicochemical properties, including molecular weight, degree of crystallinity, mechanical strength, and biodegradability [22,23,24,25,26,27]. After this, the search for microorganisms capable of synthesizing copolymer PHAs was widely deployed in many countries.

Copolymers P(3HB-*co*-3HV), formed from monomers of 3-hydroxybutyrate and 3-hydroxyvalerate, became the second most actively studied type of PHA after P(3HB). These copolymers are isodimorphic due to the cocrystallization of 3-hydroxybutyrate and 3-hydroxyvalerate monomers in morphologically similar crystal lattices—P(3HB) or P(3HV) [28,29]. It has been shown that monomers of 3HV can crystallize in either P(3HB) or P(3HV) lattices, depending on their content in the copolymer. When the content of 3HV monomers is below 37 mol.%, they crystallize in the P(3HB) lattice [30]; at higher proportion of these monomers in the copolymer, they are included in the P(3HV) lattice [28,31]. The ratio of 3HB to 3HV monomers affects the crystallization process and the degree of crystallinity of the P(3HB-*co*-3HV) copolymer. It has been shown across a large array of samples that the presence of 3HV monomers significantly affects the characteristics of the material, reducing its melting point and crystallinity. In general, the degree of crystallinity of the copolymer is lower than that in P(3HB), and it varies widely depending on the content of 3HV monomers, ranging from 39 to 69% [32].

The physical properties of a PHA are closely related to its chemical composition and degree of crystallinity, which depends on the ratio of amorphous to crystalline phases. Therefore, being able to regulate the composition of monomers and the kinetics of crystallization of PHAs is of crucial importance for the processing of these polymers and the properties of the products. Important results were obtained by colleagues from Australia headed by Professor B. Laycock, who, studying the synthesis of P(3HB-*co*-3HV) copolymers and their properties, showed that the distribution of 3HV monomers in the C-chain of 3-hydroxybutyrate can be statistically random, as well as block. This affects the conditions and rate of crystallization and the morphology of the spherulites formed, which ultimately determines the crystallinity and properties of this copolymer [33,34,35,36,37]. Thus, the 3HB to 3HV monomer ratio in P(3HB-*co*-3HV) affects the physical properties of the copolymer and products made from them, which, compared to the P(3HB) homopolymer, are more elastic, resilient, and convenient for processing [38,39].

Following these data, the search for microorganisms and biosynthesis conditions that maximize the inclusion of 3HV monomers in the 3-hydroxybutyrate chain was conducted worldwide. Research showed that for the synthesis of these copolymers in the nutrient medium, in addition to the main carbon source, it is necessary to have 3-hydroxyvalerate monomers of short-chain fatty acids (propionic or valeric) as precursors, which are usually toxic to bacteria. Over a relatively short period of time (from the 1990s to the early 2000s), extensive data were obtained showing that bacteria of various taxa are capable of synthesizing P(3HB-*co*-3HV), the 3HB to 3HV monomer ratio in the copolymer can vary widely, and this is due to the physiological and biochemical characteristics of the strains and cultivation conditions, primarily the conditions of carbon nutrition. The processes of biosynthesis of P(3HB-*co*-3HV) with the content of 3HV monomers from 0.1–0.2 to 85.0 mol.% were implemented not only by using sugars (fructose, glucose) and sugar-containing hydrolysates, but also in autotrophic conditions on CO_2_ and on heterotrophic substrates (acetate, fatty acids, glycerol, various vegetable oils, alcohols, and waste) [40,41,42,43,44,45,46,47,48,49,50,51,52,53,54,55,56,57].

The first industrial production of P(3HB-*co*-3HV) was established by ICI (UK) in 1980 with a capacity of 5000 tons per year, but due to the high cost of the product (USD 7–8 per kg), it was closed in 1999. In 2009, Telles built a plant in China with an annual capacity of P(3HB-*co*-3HV) up to 50,000 tons; Industrial SA organized the production of this copolymer using *Cupriavidus necator* bacteria, sugar-containing waste as the main substrate and propionate as a precursor. Currently, production of marketable P(3HB-*co*-3HV)-based products is limited to small-scale projects including YPACK (European Union Horizon 2020 project), and ongoing commercial production of P(3HB-*co*-3HV) is carried out as feedstock including ENMAT™ Y1000P produced by Tianan Biologic Materials Co. (Ningbo, China) [6].

Research aimed at increasing the efficiency of P(3HB-*co*-3HV) synthesis is still relevant at present [57,58,59]. Today, the key problem of P(3HB-*co*-3HV) biotechnology, as well as PHAs in general, is the high cost of the polymer, which is several times higher than that of polyolefins [60]. The cost of these promising bioplastics can be reduced and their availability increased by using waste as a carbon substrate, as well as finding effective producers and increasing the productivity of biotechnological processes in general.

Promising PHA producers, which are thoroughly studied and exploited for industrial production of these bioplastics, are strains of *Cupriavidus necator* (former names of the taxon are *Wautersia*, *Ralstonia*, *Alcaligenes*). These microorganisms, possessing a strong intracellular PHA synthesis system and a wide organotrophic potential, are capable of synthesizing PHAs of various chemical compositions under autotrophic conditions and using many heterotrophic substrates, including industrial waste. The use of waste is a way to reduce the cost of carbon substrate in biotechnology, as well as a solution to environmental problems of waste reduction in the biosphere. This contributes to the modern concept of transition from a linear to a circular economy [61,62,63].

In the most recent years, large-tonnage waste fish oil (WFO) has been considered as a substrate for the biosynthesis of target products, including PHAs. Recent publications have demonstrated the potential for synthesizing PHAs from WFO derived from the waste of the fish processing industry, including various types of fish and methods of extracting fat, and canning plant effluents [64,65,66,67,68,69,70,71,72]. There are data showing that, when growing bacteria on WFO as the main C-substrate, it is possible to synthesize not only homogeneous poly(3-hydroxybutyrate), but also copolymers with small inclusions of monomers other than 3HB [70], including P(3HB-*co*-3HV) copolymers [66]. However, data on the properties of PHA synthesized on WFO are few and very fragmentary. This determined the purpose of the present work, aimed at assessing the efficiency of the synthesis of P(3HB-*co*-3HV) copolymers using WFO as the main C-substrate and potassium valerate as a precursor of 3HV monomers, and studying how the use of such an unusual carbon source affects the ratio of monomers in the copolymer and its physicochemical properties.

## 2. Materials and Methods

### 2.1. Strain and Cultivation Conditions

The wild-type *C. necator* B-10646 strain, registered in the VKPM [73], was used in the present study.

The bacteria were grown in 0.5-L flasks containing 200 mL of Schlegel medium of the following composition (g·L^−1^): KH_2_PO_4_—1.5, Na_2_HPO_4_—9.0, Fe_3_C_6_H_5_O_7_—0.025, MgSO_4_—0.2, and a solution of trace elements (g·L^−1^): H_3_BO_3_—0.228, CoCl_2_·6H_2_O—0.03, CuSO_4_·5H_2_O—0.008, MnCl_2_·4H_2_O—0.008, ZnSO_4_·7H_2_O—0.176, NaMoO_4_·2H_2_O—0.05, NiCl—0.008, added to the medium in an amount of 3 mL/L. Bacteria were cultured in the PHA synthesis mode with a reduced NH_4_Cl concentration in the medium (0.7 g·L^−1^). Bacteria were cultured in a shaker incubator (Incubator Shaker Innova, New Brunswick Scientific, Edison, NJ, USA) at 30 °C and 200 rpm for 48 h. WFO was added to the flasks at a concentration of 20 g·L^−1^. Potassium valerate was used as a precursor of 3HB monomers. The precursor was added to the bacterial culture periodically to avoid inhibition of bacterial growth. A single addition of the precursor ranged from 0.5 to 2.0 g·L^−1^, and the total concentration ranged from 0.5 to 4 g·L^−1^. Butyric acid at a total concentration of 4.0 g·L^−1^, as the main carbon source, was used as the control (C_4_H_8_O_2_) (Acros Organics, Geel, Belgium).

### 2.2. Waste Fish Oil (WFO)

The waste from the production of canned sprats, which was produced during the processing of Baltic sprat (*Sprattus sprattus balticus*), was studied as the main carbon substrate for the synthesis of P(3HB-*co*-3HV) by an enzymatic method, using the proteolytic enzyme Alcalase^®^ 2.4 L (Novozymes, Bagsværd, Denmark) [74]. Determination of protein was carried out according to Lowry [75]. The carbohydrate content was determined by the anthrone method [76]. The lipid content was determined gravimetrically with chloroform extraction [77]. The fatty acid composition of the oils was determined after methyl esterification with sulfuric acid:methanol = 1:20 solution, which lasted 2 h at 80 °C. FAMEs were analyzed using a GC–MS 7890/5975C (Agilent Technologies, Santa Clara, CA, USA). The conditions of chromatography were as follows: helium as a carrier gas with a flow rate of 1 mL min^−1^; injector temperature of 220 °C; initial temperature of 120 °C; elevation of temperature to 230 °C at a rate of 5 °C min^−1^ with 5 min of isothermal regime and subsequent elevation of temperature to 310 °C at a rate of 10 °C min^−1^ with 3 min of isothermal regime; interface temperature of 230 °C; ion source temperature of 150 °C; electron impact at 70 eV; scanning of the fragments with atomic masses from 30 to 550 amu at 0.5 s scan^−1^. The FAs were identified by comparing their retention times and mass spectra to the known standards.

### 2.3. The Parameters of the Bacterial Growth Process

The cell concentration was assessed by drying a twice-washed biomass at 105 °C for 24 h, which was centrifuged at 6000 rpm. The polymer content in bacterial cells and the polymer composition were determined using a gas chromatograph with a mass spectrometer (7890A/5975C Agilent Technologies, Santa Clara, CA, USA). For this fatty acid, methyl esters were obtained as described previously [78]. To correctly determine the ratio of monomers and the content of 3HV monomers in the copolymer, NMR spectroscopy was used. ^1^H NMR spectra of PHA samples were recorded at room temperature in CDCl_3_ in a Bruker Avance III 600 spectrometer (Bruker, Rheinstetten, Germany) operating at 600.13 MHz. IR spectra were taken in the 400–4000 cm^−1^ range using a “NICOLET 6700” FT-IR spectrometer (Thermo Scientific, Waltham, MA, USA) and a Smart Orbit accessory, using the attenuated total reflection (ATR) technique.

The process parameters were cell biomass yield (X, g·L^−1^), intracellular polymer content (% of DCW), biomass (P_X_) and polymer (P_PHA_) productivity (g·L^−1^·h^−1^):

P_X_ = (X_final_ − X_0_)/t_total_, (1)

P_PHA_ = (PHA_total_ − PHA_0_)/t_total_, (2)

where PHA_total_, PHA_0_ are the final and initial concentrations of polymer in cells, g·L^−1^.

### 2.4. Polymer Properties

Extraction and purification of polymer samples were described previously [78]. The molecular weight characteristics of the PHA samples were determined using size-exclusion chromatography (Agilent Technologies 1260 Infinity, Waldbronn, Germany; Agilent PLgel Mixed-C column). Chloroform was used as a solvent, and a set of polystyrene standards (Agilent Technologies, Santa Clara, CA, USA) was used as standards.

The melting point was determined from the exothermic peaks in the thermograms (STARe software, version 11.0) using a DSC-1 differential scanning calorimeter (Mettler Toledo, Schwerzenback, Switzerland); the thermal degradation temperature was determined by the TGA2 (Mettler Toledo, Schwerzenback, Switzerland). The theoretical degree of crystallinity (C_x_, the content of the crystalline phase expressed as a percentage) was calculated using the formula:

C_x_ = (∆H_i_)/(∆H_0_), (3)

where ∆H_i_ is specific enthalpy of melting of the sample (J/g); ∆H_0_ is specific enthalpy of melting of 100% crystallized P(3HB), 146 J/g [79].

Crystallization (crystallization time—time interval from the beginning to the end of the formation of the crystalline phase in the sample; crystallization rate—change in sample crystallinity per unit time) of the samples was studied in the isothermal mode. The samples were heated to 200 °C, held for 3 min, and then cooled to the crystallization temperature (70, 80, 90, 100 °C) at a rate of 50 °C/min. The samples were held for 45 min and then heated to 200 °C. The degree of crystallinity was determined by the enthalpy of melting of the formed crystalline phase. All experiments were carried out in a nitrogen atmosphere.

### 2.5. Study of Spherulite Morphology

The morphology and radial growth rate of spherulites of P(3HB-*co*-3HV) samples synthesized on WFO and butyric acid and subjected to isothermal crystallization were observed using a polarizing optical microscope (Nikon, Eclipse E600 POL, Tokyo, Japan), a 10× magnification (field of view: 22 mm), equipped with a hotstage (Linkam, LTS420, Salfords, UK). To destroy the thermal history, the samples were melted at 195 °C for 3.5 min and then cooled to the desired temperature. The spherulitic growth rate (G) was calculated from the change of radius (R) with time (t). Observation was continued until the field of view was completely covered by spherulites.

### 2.6. Statistics

All experiments were performed in three biological replicates. Statistical analysis was carried out using the standard Microsoft Excel software package. Arithmetic means and standard deviations (SDs) were found (mean ± SD). The statistical significance of the results was determined using Student’s *t*-test (significance level: *p* ≤ 0.05). Two-factor analysis of variance (ANOVA) was used (significance level: *p* ≤ 0.05) to evaluate the effect of the type of carbon substrate (WFO, butyric acid) and the concentration of the precursor potassium valerate on the production parameters of bacterial culture.

## 3. Results

The waste from the production of canned sprats, which was produced during the processing of Baltic sprat (*Sprattus sprattus balticus*), was studied as the main carbon substrate for the synthesis of P(3HB-*co*-3HV). The WFO samples contained fat (total lipids), carbohydrates, and protein: 89.2 ± 1.8, 1.8 ± 0.1, and 2.6 ± 0.2 (% of absolutely dry matter), respectively. The fatty acid composition of the WFO is shown in Table S1. Twenty-four fatty acids with a C-chain length of 14 to 24 carbon atoms were identified in the WFO. The main FAs were palmitic (25.05%), oleic (28.09%), docosahexaenoic (13.71%), and eicosapentanoic (9.08%) FAs. The content of saturated stearic and myristic, as well as polyene linoleic and linolenic fatty acids was 1.48–4.95%. The content of long-chain monoene fatty acids (C20–C24) was 1.19–2.24%. The ratio of saturated to unsaturated fatty acids was determined at the level of 0.50.

### 3.1. Synthesis of P(3HB-co-3HV) Using Waste Fish Oil as the Main Carbon Substrate

The possibility of the synthesis of P(3HB-*co*-3HV) copolymers and its parameters were studied in the culture of the wild-type strain *C. necator* B-10646, characterized by the ability to synthesize PHA copolymers of various chemical compositions, including P(3HB-*co*-3HV). As shown previously, depending on the amount of potassium valerate additives in the culture of these bacterial cells grown on glycerol of varying purity, the content of 3HV monomers in the copolymer ranged from 29 to 36 mol.% [80]; on glucose from 10 to 65 mol.% [81]; and in an autotrophic culture on a gas mixture of CO_2_/O_2_/H_2_, it was up to 85 mol.% [82].

**Figure 1:**
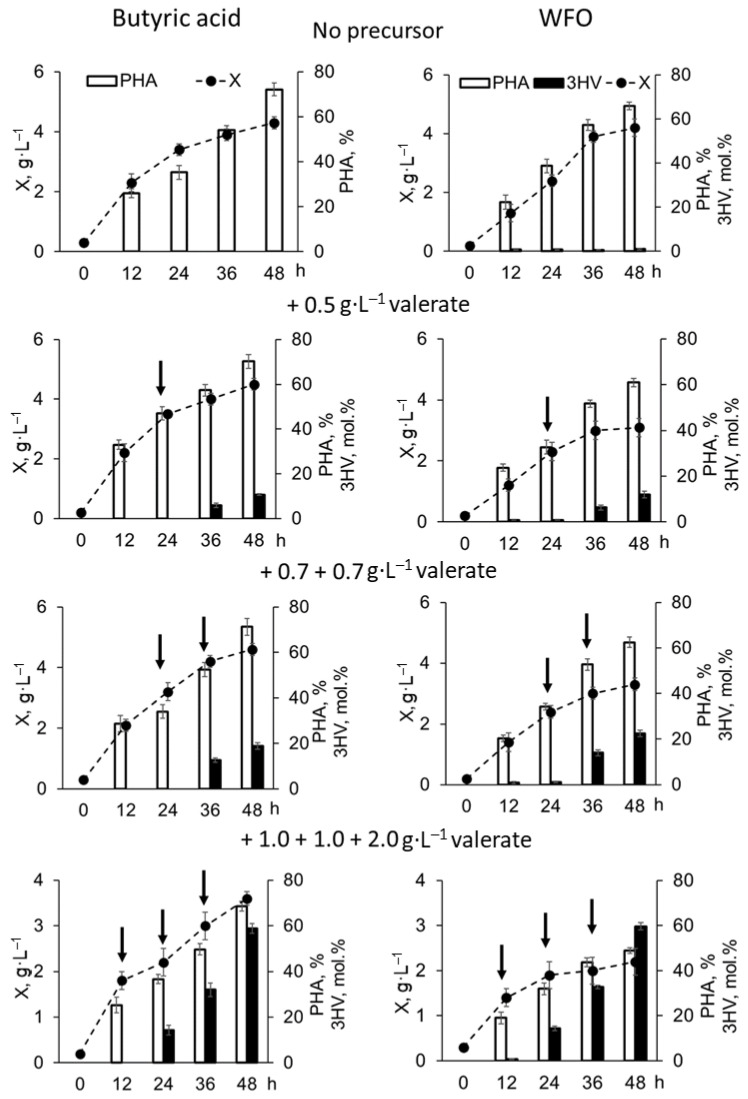
Parameters of the batch culture of *C. necator* B-10646 grown on WFO as the main C-substrate with the addition of potassium valerate relative to the control (butyric acid): total cell biomass yield, X (g·L^−1^), intracellular content of P(3HB-*co*-3HV), % of CDW; content of 3HV monomers in the copolymer (mol.%). Arrows point at the addition of potassium valerate.

**Figure 2:**
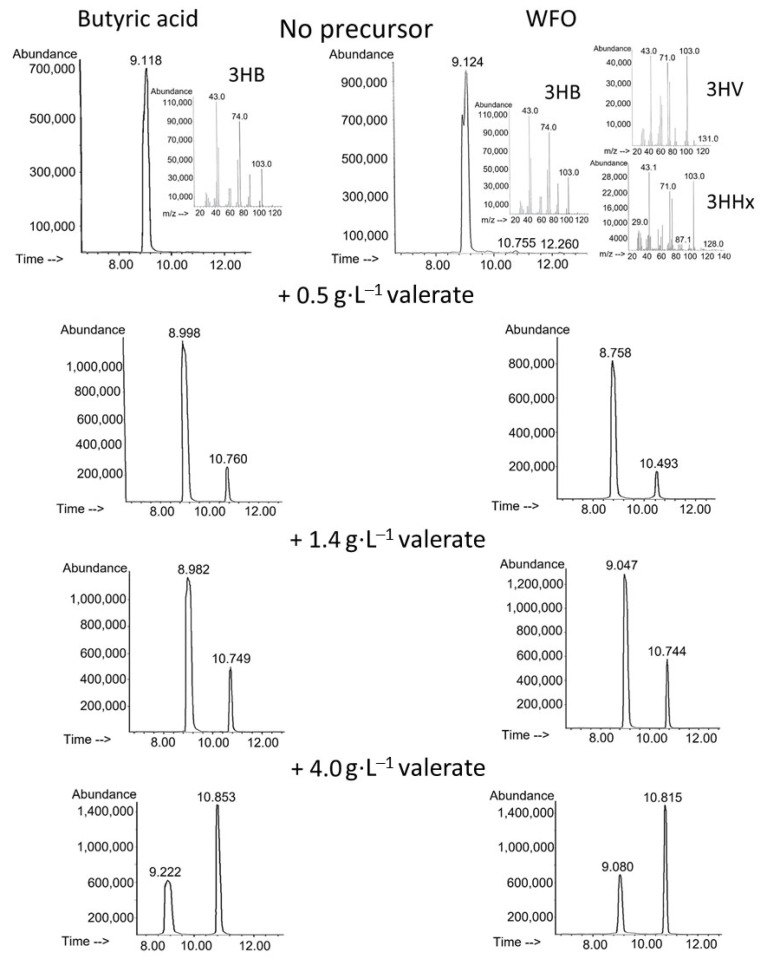
Chromatograms and mass spectra of methyl esters of PHA samples synthesized on WFO and butyric acid with different ratios of 3HB to 3HV monomers. The retention time of the 3HB unit is 8.758–9.222 min; the 3HV unit—10.493–10.853 min; the 3HHx—12.260 min.

Synthesis of P(3HB-*co*-3HV) copolymers with significant proportions of 3-hydroxyvalerate monomers by *C. necator* B-10646 cells on WFO was studied for the first time. The parameters of the batch culture of *C. necator* B-10646 cells grown using WFO in the PHA synthesis mode with limitation of bacterial growth by nitrogen and different amounts of potassium valerate additives are compared with the control (butyric acid) in Figure 1. Chromatograms of a series of P(3HB-*co*-3HV) samples and mass spectra of the corresponding methyl esters of the monomers illustrate different ratios of 3-hydroxybutyrate to 3-hydroxyvalerate monomers in the synthesized copolymer samples (Figure 2).

Potassium valerate was used as a precursor of 3-hydroxyvalerate monomers, which was added to the culture medium in split portions to avoid inhibition of bacterial growth. The following dosing regimens of the precursor to the bacterial culture were used: a single addition for 24 h of bacterial growth at a concentration of 0.5 g·L^−1^; two-fold addition for 24 and 36 h at 0.7 g·L^−1^ (a total of 1.4 g·L^−1^); three-fold addition for 12, 24 and 36 h at 1.0, 1.0 and 2.0 g·L^−1^, respectively (a total of 4.0 g·L^−1^). The duration of the bacterial cultivation process did not exceed 48 h. The results of culturing the wild-type strain *C. necator* B-10646 and the synthesis of P(3HB-*co*-3HV) during growth on a complex fat-containing substrate (WFO) and butyric acid as the control are shown in Figure 1 and Figure 2 and in Table 1. Successful experiments with butyric acid used as the main C-substrate for the synthesis of P(3HB-*co*-3HV) copolymers are described in a number of publications. Thus, in bacterial cultures of various strains (*A. eutrophus* NCIB 11599, *R. eutropha* DSM 428, *Haloferax mediterranei*), depending on the bacterial cultivation conditions (in flasks or in a fermenter), as well as on the type and dosage regimen of 3-hydroxyvalerate monomer precursors, the intracellular content of the copolymer varied from 37% to 80%, and the content of 3HV monomers in the copolymer varied from 9 to 85 mol.% [46,83,84,85].

The results of the chromatographic analysis of the PHA samples (Figure 2) showed that when bacteria grew on butyric acid without potassium valerate added to the culture, the synthesized polymer was a homopolymer of 3-hydroxybutyric acid. When WFO was used as a carbon substrate, a three-component copolymer was synthesized, which, in addition to the dominant monomers of 3-hydroxybutyrate, contained minor inclusions of the monomers 3-hydroxyvalerate (1.0 mol.%) and 3-hydroxyhexanoate (0.4 mol.%). The reason for this is that WFO is a carbon substrate of complex composition, in which a set of free fatty acids of various structures is formed from triacylglycerols as a result of the lipolytic activity of bacterial cells. Among the studied WFOs, there are saturated, unsaturated, mono- and polyenoic FAs (Table S1). Apparently, individual fatty acids (with even or odd C-chain length) are capable of acting as precursors of monomers other than 3HB: 3HHx or 3HV monomers, respectively. At the same time, the total cell biomass yield and intracellular copolymer content produced on WFO and butyric acid were comparable: 4.2–4.3 g·L^−1^ and 66–71%, respectively.

The addition of potassium valerate to the bacterial culture was accompanied by an increase in the content of 3HV monomers in the copolymer (Figure 1 and Figure 2). At the same time, depending on the dose of potassium valerate, the biomass yield and total copolymer yield changed differently depending on the main carbon source used. In the control (on butyric acid), the inhibitory effect on the value of X (g·L^−1^) was manifested only at the maximum dose of the precursor (three additions of potassium valerate, total concentration of 4.0 g·L^−1^), when the bacterial biomass concentration slightly decreased, to 3.6 ± 0.1 g·L^−1^. During cell cultivation on WFO, the inhibitory effect of potassium valerate was manifested even at its lowest concentration (one addition, 0.5 g·L^−1^) and became more pronounced as it increased. The bacterial biomass yield decreased considerably, to 2.2 ± 0.3 g·L^−1^, when three portions of potassium valerate (total concentration of 4.0 g·L^−1^) were added.

The intracellular content of the copolymer in *C. necator* B-10646 cells grown on WFO with different dosing regimens of potassium valerate in the culture (one, two and three additives) was 61 ± 2, 63 ± 2 and 49 ± 2%, respectively. That was inferior to the results in the control (bacterial growth on butyric acid) with similar precursor addition regimens, when the copolymer content in the cells was higher: 70 ± 3, 71 ± 4 and 67 ± 3%, respectively (Table 1).

The use of the two studied C-substrates did not affect the 3HB to 3HV monomer ratio in the copolymer, in contrast to the total yield of the copolymer (Figure 2). With one addition of the precursor, the contents of 3HV monomers in the copolymer produced on WFO and butyric acid did not differ significantly, 11.9 ± 0.4 and 10.7 ± 0.4 mol.%; with two additions, they were 22.2 ± 1.5 and 18.9 ± 0.4 mol.%, respectively. Even with three additions of potassium valerate to the culture, when an inhibitory effect on the total bacterial yield and total copolymer yield was noted, the content of 3HV monomers did not differ significantly in the experiment on WFO and in the control on butyric acid, 59.7 ± 3.1 and 58.8 ± 2.1 mol.%, respectively. The polymer synthesized on the fat substrate under all precursor addition conditions was a two-component copolymer P(3HB-*co*-3HV), and no 3-hydroxyhexanoate monomers (3HHx) were found in it, even in trace amounts. This is a difference from the result of PHA synthesis by *C. necator* B-10646 cells on the WFO type studied here (fat from smoked sprat heads) without the use of a precursor, obtained in this work and previously, which showed that on this type of fat waste, bacteria of this strain synthesize a three-component copolymer—P(3HB-*co*-3HV-*co*-3HHx) [70,86]. It can be assumed that when bacterial cells are grown on WFO of complex composition, represented by a set of fatty acids of different structures, and another component—potassium valerate—is added to the medium as a precursor of 3HV monomers, its preferential transport into the cell, metabolism, and inclusion in the C-chain of the synthesized polymer in the form of 3-hydroxyvalerate monomers are sure to occur. At the same time, under these conditions, other FAs in the WFO, which are capable of acting as precursors of 3-hydroxyhexanoate monomers, may be inferior to valerate, and, for some reason, their transport into the cell may be hindered. However, this issue requires an additional and special study. Moreover, the aim of the current study was to synthesize P(3HB-*co*-3HV) copolymers, and it was achieved.

**Figure 3:**
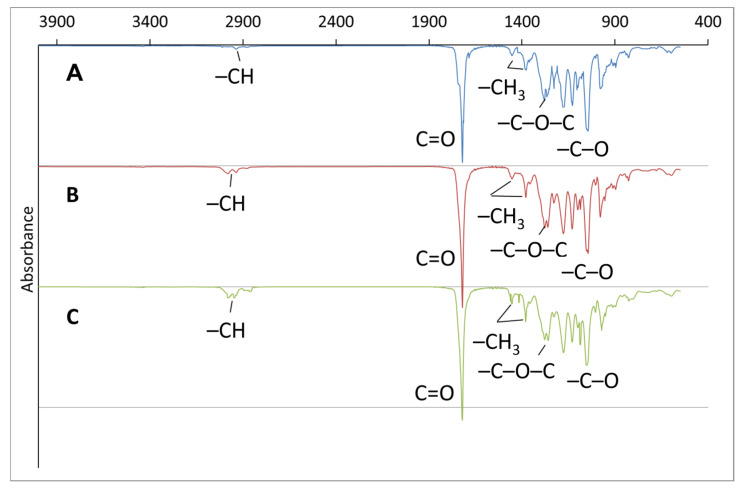
IR spectra of P(3HB-*co*-3HV) synthesized by *Cupriavidus necator* B-10646 grown on WFO with different contents of 3HV monomers: 11.9 mol.% (A); 22.2 mol.% (B); 59.7 mol.% (C).

IR Fourier spectroscopy was used to study the qualitative composition of the copolymers (Figure 3). The absorption peak at 1278–1282 cm^−1^ was attributed to the absorption of the ester bond C–O–C. The absorption peaks at 1375 and 1450 cm^−1^ were attributed to the stretching and deformation vibrations of the methyl group (–CH_3_). The intense peak at 1720 cm^−1^ was attributed to vibrations of the carbonyl group C=O. The double peak at 1950 cm^−1^ was attributed to vibrations of the methine (C–H) group [31,87].

**Figure 4:**
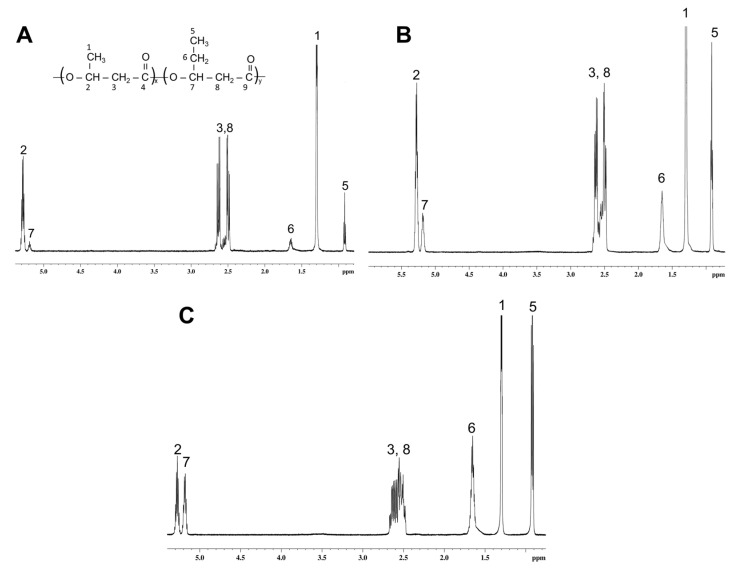
^1^H NMR spectra of P(3HB-*co*-3HV) synthesized by *Cupriavidus necator* B-10646 grown on WFO with different contents of 3HV monomers: 11.9 mol.% (**A**); 22.2 mol.% (**B**); 59.7 mol.% (**C**).

The additional methyl group in the copolymer introduces a difference in the IR spectrum from the spectrum of the homopolymer, which is expressed in additional vibrations of stretching and bending of the CH group in the region of 2850–3000 cm^−1^ and the region of 1380–1460 cm^−1^. In the study in [88], the intensity of the absorption bands at 1084 cm^−1^, 1050 cm^−1^, 1004 cm^−1^ and 968 cm^−1^ is related to the composition of P(3HB-*co*-3HV). Our studies confirm an increase in the intensity of the absorption bands in the indicated regions with an increase in the 3HV content. However, the IR spectra of the copolymers do not provide a definite answer concerning their composition. There are discrepancies in the interpretation of the spectra. Thus, in some studies, absorption at 1050 cm^−1^ is attributed to symmetric stretching of the C–O bond [89], and in others to bending of the C–CH_3_ bond [90]. The same is observed for the absorption band at 968 cm^−1^ [90,91]. Therefore, an NMR study was carried out to specify the composition of the synthesized samples (Figure 4).

The productivities of the *C. necator* B-10646 bacterial culture synthesizing PHA on WFO and butyric acid without a precursor were similar to each other in terms of cell biomass and polymer yield. However, when the precursor was added to the medium in the P(3HB-*co*-3HV) copolymer synthesis mode, these parameters changed differently (Table 1). In the control on butyric acid, both parameters (P_X_ and P_P(3HB/3HV)_) slightly decreased only with three additions of valerate to the culture. In the culture with WFO used as the main carbon substrate, a decrease in productivity in terms of total biomass and copolymer yield was recorded for all three precursor dosing modes. A particularly noticeable decrease was observed in the treatment with three potassium valerate additions; the productivity in terms of total yield decreased by a factor of 1.8 and, in terms of copolymer production, by a factor of 2.5 compared to the corresponding parameter without a precursor. The reason for that was the toxicity of the precursor for cells, despite the portioned addition of potassium valerate to the culture.

**Figure 5:**
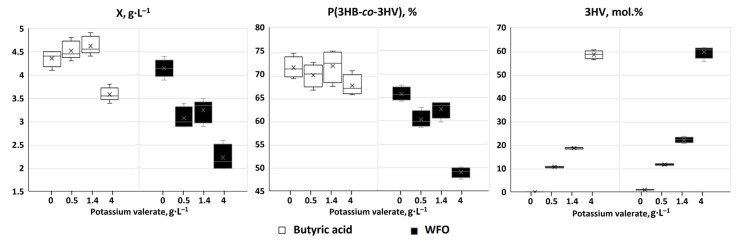
Results of two-way analysis of variance (ANOVA) of production parameters of the *C. necator* B-10646 bacterial culture grown on WFO compared to butyric acid (control): total yield, X, g·L^−1^, intracellular content of copolymer, % of CDW, content of 3HV monomers in the copolymer, mol.%.

These experiments demonstrated for the first time the possibility of synthesizing P(3HB-*co*-3HV) copolymers with significant proportions of 3HV monomers with satisfactory total cell biomass yields and intracellular copolymer contents, using renewable sprat production waste as the main carbon substrate. The effect of potassium valerate as a precursor of 3HV monomers on the bacterial culture parameters was different depending on the type of the main carbon substrate (WFO or butyric acid). Processing of the results using two-way analysis of variance (ANOVA) showed that the carbon substrate, precursor concentration, and the interaction of these factors had a significant effect on the total biomass yield and polymer content, which were higher in the samples synthesized on butyric acid compared to WFO (Figure 5, Table S2).

The type of C-substrate and the concentration of the precursor had a significant effect on the biomass yield (*p* < 0.05), with the effect of the precursor being less pronounced than the effect of the main substrate. A significantly higher X, g·L^−1^, value was obtained with butyric acid with the addition of a total concentration of potassium valerate of 1.4 g·L^−1^ and with WFO without the addition of the precursor. The interaction of the factors (C-substrate + precursor concentration) had a significant effect (*p* < 0.05) on the biomass yield. The copolymer content in the cells significantly depended on the type of C-substrate, with both factors and their interaction having a significant effect (*p* < 0.05) on the copolymer content. Higher copolymer content was found without the addition of the precursor and the lowest ones were recorded with the maximum concentration of potassium valerate (4.0 g·L^−1^). The precursor concentration had a significant effect on the content of 3HV monomers in the copolymer (*p* < 0.05); with an increase in the concentration of potassium valerate, the content of 3HV monomers in the copolymer increased. The type of C-substrate had a significant, but less important effect, (*p* < 0.05). The interaction of substrate and precursor concentrations did not have a significant effect on the content of 3HV monomers (*p* = 0.44).

A detailed comparison of the obtained results with the literature data on the efficiency of using WFO as the main substrate for the synthesis of P(3HB-*co*-3HV) is difficult because there are very few studies on the synthesis of P(3HB-*co*-3HV) using fish processing fatty waste as the main carbon substrate. Two articles on this subject were found in the available literature. In the work by colleagues from Vietnam, the synthesis of PHA on various substrates in the culture of the halogen-resistant wild-type strain *Salinivibrio* sp. M318 was studied. That study showed that on mixtures of WFO from Basa fish (*Pangasis bocourti*) waste and glycerol using potassium valerate, propionate or hexanoate as precursors, it is possible to synthesize P(3HB-*co*-3HV) copolymers with a 3HV monomer content from 13.3 to 24.7 mol.% with a total copolymer yield of about 48–53% [68]. The possibility of synthesizing P(3HB-*co*-3HV) is described in [66]. Using the repeated-batch cultivation of *Cupriavidus necator* TISTR cells and WFO fat condensate from tuna processing, a copolymer with a 3HV monomer content of 20 mol.% was synthesized. Thus, in the present work, higher results were obtained; with a total copolymer yield of up to 60–70%, the inclusion of 3HV monomers was highest, 59.7 ± 3.1 mol.%. A series of P(3HB-*co*-3HV) samples with 3HV content from 10 to almost 60.0 mol.% was synthesized. This allowed us to study the effect of the 3HB to 3HV monomer ratio on the properties of the copolymer, including the yields of the copolymer and the composition of the monomers depending on the type of the C-substrate used (WFO of complex composition with a set of fatty acids of different structures and a single compound—butyric acid).

### 3.2. Physicochemical Properties of P(3HB-co-3HV) with Different Ratios of 3HB to 3HV Monomers Synthesized on WFO or Butyric Acid

PHAs constitute a family of polymers of various chemical structures synthesized by prokaryotes. The basic properties of these polymers are determined by the composition and proportions of monomers in them. In turn, the possibility of synthesizing PHAs of various chemical compositions depends on the physiological and biochemical specificity of producer strains and metabolic pathways of the PHA cell cycle, as well as on the conditions of bacterial cultivation, primarily on the conditions of carbon nutrition. The physicochemical properties of polymer samples with various sets and ratios of monomers synthesized by the wild-type strain *C. necator* B-10646 on WFO as the main carbon source are shown in Table 2, which also presents the composition and properties of the samples synthesized in the control (on butyric acid).

The molecular weight characteristics of the three-component sample synthesized on WFO were similar to those of the P(3HB) homopolymer sample synthesized on butyric acid. The number-average (M_n_) and weight-average (M_w_) molecular weights of the polymers synthesized on butyric acid and WFO were 190 and 219 kDa and 418 and 528 kDa, respectively, and the polydispersity was 2.2 and 2.4. This differs from the data for P(3HB) synthesized on fructose and vegetable oils reported in our previous study. With a similar number-average molecular weight of 130–220 kDa, the weight-average molecular weight on these substrates was considerably higher, 670–750 kDa, and the polydispersity was 3.4–5.2 [92].

The PHA samples synthesized on WFO with different additions of potassium valerate contained monomers of 3-hydroxybutyrate and 3-hydroxyvalerate in amounts comparable with those in the control samples (butyric acid).

**Figure 6:**
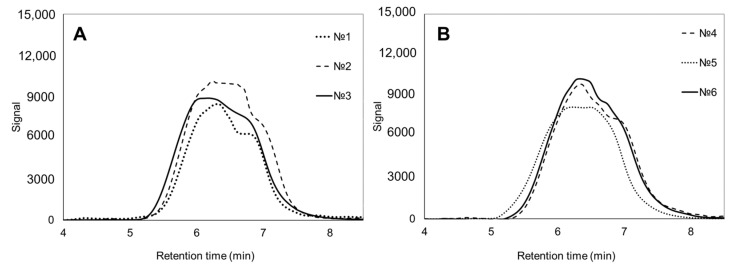
GPC traces of PHA samples synthesized by *Cupriavidus necator* B-10646 on WFO (**B**) and butyric acid (**A**) with different additions of potassium valerate. Sample numbering according to Table 2.

Changes in the molecular weight of samples with 3HV obtained on butyric acid and WFO followed different trends. For instance, in the first case, the samples with 3HV showed an increase in M_w_ and polydispersity to 430–520 and 2.4–2.7, respectively. Polydispersity increased with the amount of the precursor added to the medium, and, consequently, with an increase in the 3HV content in the copolymer. Similar to the samples obtained on butyric acid, the synthesis of copolymers with 3HV on WFO was also accompanied by an increase in polydispersity, which reached 2.6–3.0. However, no direct relationship was found between polydispersity and the added precursor concentration and, consequently, the 3HV content. As for M_n_ and M_w_, with the exception of the copolymer containing 22.2 mol.% of 3HV, the other two copolymer samples showed a decrease in both M_n_ and M_w_ to 130–142 and 390–417 kDa, respectively (Figure 6).

There are very few studies of P(3HB-*co*-3HV) synthesized on WFO. The molecular weight characteristics of the copolymer samples synthesized by *Salinivibrio* sp. grown on WFO mixtures from Basa fish (*Pangasius bocourti*) waste and glycerol with the addition of several precursors of 3HV monomers were studied in [64]. The authors showed that for the copolymer samples with a 3HV monomer content of 13.3, 17.0, and 24.7 mol.%, the M_w_ values were 200, 630, and 530 kDa; the M_n_ values were 120, 320 and 310 kDa, respectively; polydispersity was 1.7, 2.0 and 1.7. Thus, with an increase in the content of 3HV monomers, the molecular weight of the copolymer increased. Those data differ from the result obtained in the present work. Another value of the molecular weight of P(3HB-*co*-3HV) is given in [66]. The copolymer synthesized on the condensate from tuna fat waste and containing 20 mol.% of 3HV monomers had extremely low M_w_ and M_n_ values, 5.0 and 2.0 kDa, respectively, with a polydispersity of 2.5.

**Figure 7:**
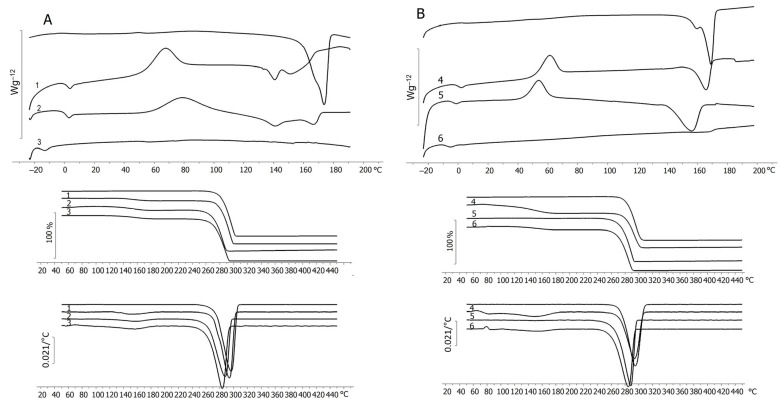
Thermal characteristics (DSC, TGA) of PHA samples with different monomer contents synthesized by *C. necator* B-10646 on butyric acid (control) (**A**) and WFO (**B**). Sample numbering according to Table 2.

The results of measuring the thermal characteristics of the P(3HB-*co*-3HV) samples synthesized on WFO, as well as in the control on butyric acid, with different and similar monomer ratios, revealed the following differences (Table 2, Figure 7).

Homopolymer P(3HB) synthesized on butyric acid had a higher melting point (T_melt_) (173.2 ± 0.2 °C) than the sample synthesized on WFO (168.7 ± 0.1 °C). It was not possible to record the glass transition temperature (T_g_) of the P(3HB) sample synthesized on butyric acid. However, the thermal degradation temperatures (T_degr_) of the P(3HB) samples synthesized on butyric acid and WFO were comparable; the onset of weight loss was recorded at 281.9 ± 2.3 °C.

All copolymer samples of P(3HB-*co*-3HV), synthesized with the addition of the precursor, regardless of the type of carbon substrate, had lower melting points. As can be seen from the thermograms (Figure 7), the samples of P(3HB-*co*-3HV) with a 3HV monomer content of 10.7 and 18.9 mol.% synthesized on butyric acid contained two melting peaks in the range of 140–166 °C. The boundaries of the melting peaks are more diffuse compared to the melting peak of the homopolymer. The presence of two peaks may indicate the formation of two crystalline modifications. A decrease in the glass transition temperature with an increase in the content of 3HV monomers is characteristic of the copolymer samples. With a 3HV monomer content of 10.7 mol.% in the copolymer, the glass transition temperature was determined at 0.4 ± 0.1 °C; at 58.8 mol.%, it decreased to −16.6 ± 0.4 °C. The control samples of the copolymer with a 3HV monomer content of 58.8 mol.% did not have crystallization and melting peaks on the thermograms, which indicated the absence of their crystallization. For all samples of the copolymers synthesized on butyric acid, a small weight loss (up to 4%) was noted in the range of 120–142 °C. The highest value of the thermal degradation temperature was observed for the sample with a 3HV monomer content of 10 mol.% (280.4 ± 3.1 °C). This is comparable with the thermal degradation temperature of the P(3HB) homopolymer. The thermal degradation temperature of the copolymers with a 3HV content of 18.9 and 58.8 mol.% was significantly lower, 268.3 ± 2.5 and 271.8 ± 2.1 °C, respectively.

The samples synthesized on WFO, in contrast to the samples in the control on butyric acid, had one melting peak, which was shifted to the region of low temperatures with an increase in the content of 3HV monomers. The melting point of these samples was generally slightly lower than that of the samples in the control. Thus, the sample with the highest content of 3HV monomers (59.7 mol.%), synthesized on WFO, had a weakly expressed diffuse melting peak at 163.9 ± 0.1 °C. For that sample, the thermogram upon cooling did not show thermal effects related to crystallization. The samples synthesized on WFO, like the control samples, showed a decrease in the glass transition temperature with an increase in the content of 3HV monomers. The lowest value was recorded for the sample with a 3HV content of 59.7 mol.% (−9.0 ± 0.1 °C). Similarly, as the content of 3HV monomers increased, the thermal degradation temperature decreased to 270.4 ± 1.7 °C with a maximum content of 3HV (59.7 mol.%).

Comparison of the obtained results with the published data showed that the study of the thermal properties of P(3HB-*co*-3HV) copolymers synthesized by *Salinivibrio* sp. bacteria on mixtures of WFO (Bosa fish waste) and glycerol with the addition of 3HV monomer precursors was only found in one work [64]. The authors showed that the content of 3HV monomers affects the temperature characteristics of the P(3HB-*co*-3HV) copolymer. With a 3HV monomer content of 13.3 mol.% in the copolymer, two peaks were recorded in the melting region at 141 and 160 °C; the crystallization (T_cryst_) and glass transition temperatures of the sample were about 51.0 and 1.0 °C, respectively. Similarly, with a 3HV content of 17.0 mol.%, two melting peaks were recorded, at 132 and 150 °C; T_g_ was 60 °C; no T_g_ peak was recorded. With an increase in the 3HV monomer content to 24.7 mol.%, one melting peak was recorded on the thermogram, at 139 °C; the crystallization temperature of that sample was not determined; the glass transition temperature was at −4 °C. These results generally correspond to the data on the thermal characteristics of the P(3HB-*co*-3HV) copolymer samples synthesized by *C. necator* B-10646 cells on WFO produced during sprat production, obtained in the present work.

The C_x_ of the P(3HB-*co*-3HV) samples with different contents of 3HB and 3HV monomers synthesized by *C. necator* B-10646, determined from the DSC results, are presented in Table 2. The C_x_ value decreased depending on the content of 3HB and 3HV monomers, which is typical for PHA copolymers. Thus, the samples synthesized without a precursor, i.e., the P(3HB) homopolymer on butyric acid and the copolymer synthesized on WFO and containing, in addition to 3HB monomers, minor inclusions of 3HV and 3HHx monomers, had C_x_ of 62.0 ± 2.0 and 45.1 ± 1.0%, respectively. The samples synthesized on WFO and containing 11.9, 22.2, and 59.7 mol.% of 3HV monomers had C_x_ values of 32.2 ± 1.2, 36.5 ± 3.5, and 3.5 ± 0.9%, respectively. The control samples with 10.7 and 18.9 mol.% of 3HV monomers had C_x_ values of 50.4 ± 2.2 and 30.2 ± 2.7%. As there were no melting and crystallization peaks in the thermograms of the sample with the highest content of 3HV monomers, its C_x_ could not be determined. It was noted above, in the Introduction section, that no data on the crystallinity of P(3HB-*co*-3HV) copolymers synthesized on WFO were found in the available literature. Therefore, the results of determining the C_x_ of the copolymers synthesized on fish processing fat waste were obtained in this work for the first time.

### 3.3. Thermal Behavior and Exothermic Crystallization of P(3HB-co-3HV) Copolymers with Different Monomer Ratios Synthesized by C. necator B-10646 on WFO or Butyric Acid

The physical properties of PHAs, including their thermal behavior, are closely related to their chemical composition and degree of crystallinity, which depends on the ratio of amorphous to crystalline phases. Being able to regulate the composition of monomers and the kinetics of crystallization of PHAs is of crucial importance for the processing of these polymers by thermal methods from melts and the properties and quality of the resulting polymer products. Much attention is being paid to this aspect of polymer materials research. The process of polymer crystallization occurs during cooling of melts or during their precipitation from solutions, and it is a first-order phase transition with temperature and heat of transition values inherent in a PHA of a specific chemical composition. Melting and crystallization of copolymer PHAs depend on the type and ratio of monomers, which determine the temperature characteristics and the ratio of amorphous to crystalline phases (C_x_).

A number of papers on the thermal behavior and crystallization kinetics of PHA samples with different monomer compositions and proportions, including copolymers containing monomers of 3-hydroxybutyrate with 4-hydroxybutyrate or with 3-hydroxyvalerate or 3-hydroxyhexanoate, have been published [34,35,36,37,93,94]. At the same time, there are very few comparative studies of the thermal characteristics and the kinetics of crystallization of PHA copolymers synthesized on new promising fat substrates, including waste. In this work, for the first time, the crystallization of P(3HB-*co*-3HV) copolymers with different ratios of 3HB to 3HV monomers synthesized on WFO as a complex fat substrate derived from waste of the production of canned sprats is compared with the crystallization of the copolymer samples of similar chemical composition synthesized on butyric acid (control).

**Figure 8:**
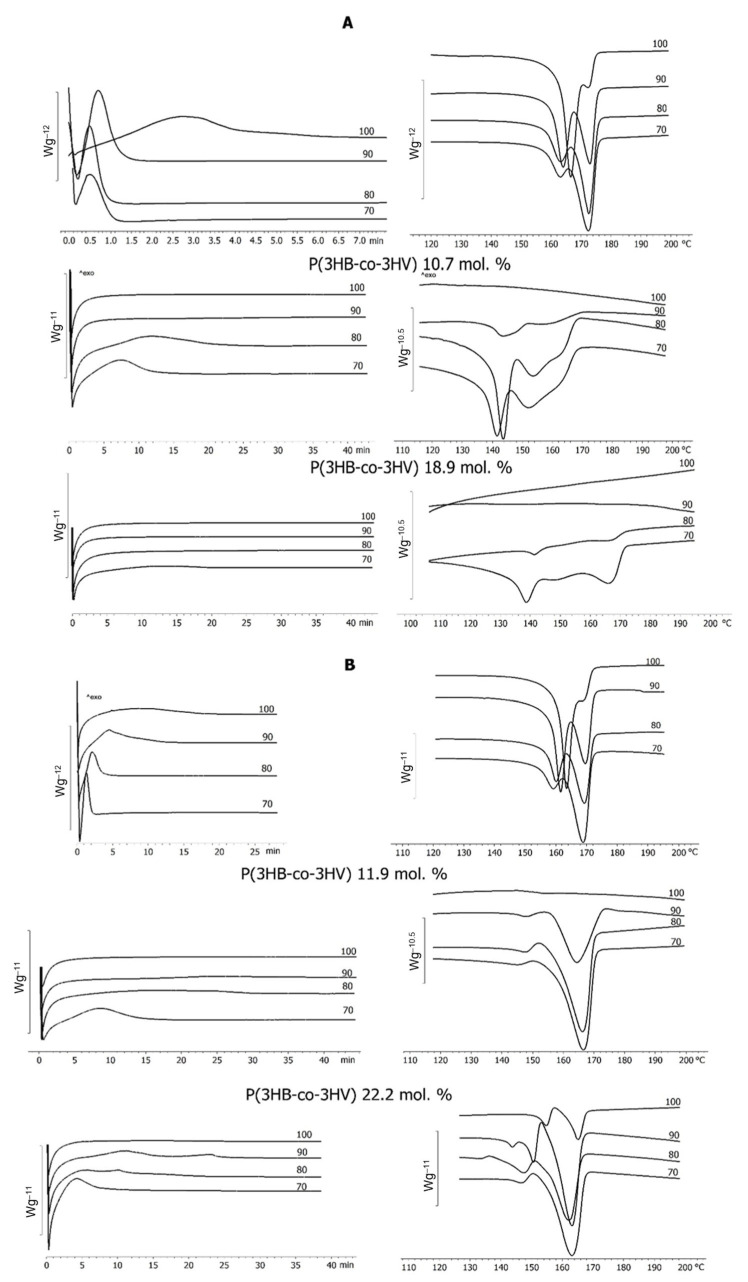
Isothermal crystallization of PHA samples with different 3HV monomer contents synthesized by *C. necator* B-10646 on butyric acid (control) (**A**) and WFO (**B**).

The isothermal crystallization kinetics of P(3HB-*co*-3HV) copolymers with different 3HB to 3HV monomer ratios was investigated by using differential scanning calorimetry (DSC) and hot-stage polarized optical microscopy (POM) (Figure 8).

The first detected effect of using WFO as the main C-substrate was the differences in the isothermal crystallization between the three-component sample synthesized on WFO without a precursor and containing minor inclusions of 3HV and 3HHx monomers and the crystallization of the P(3HB) homopolymer synthesized on butyric acid (control). The highest crystallization rates were recorded for the P(3HB) sample synthesized on WFO during crystallization at 70 °C; the crystallization time of the sample was 1.95 ± 0.15 min, and the C_x_ was 58.0 ± 3.1%. With an increase in the isothermal crystallization temperature, the crystallization peaks on the thermograms became wider, and their height decreased, which indicated an increase in the crystallization time. It took the longest time for crystallization of the sample to occur at 100 °C—26.7 ± 1.1 min; C_x_ decreased to 42.8 ± 2.4%. In contrast to the samples synthesized on WFO, the homopolymer samples synthesized on butyric acid were crystallized over a shorter time: at 70 °C—0.37 ± 0.12 min, at 80 °C—0.83 ±0.14 min, at 90 °C—1.23 ± 0.23 min, and at 100 °C—4.71 ± 0.81 min. The C_x_ value was comparable with the C_x_ value of the P(3HB) samples synthesized using WFO, and it also decreased from 56 to 52% with an increase in the crystallization temperature.

A comparative study of the exothermic crystallization of two groups of copolymers with different but comparable contents of 3-hydroxyvalerate monomers showed that the crystallization rates were generally higher for all samples synthesized on WFO (Figure 8). Thus, the sample synthesized on WFO and containing 11.9 ± 1.3% of 3HV had a crystallization time of 14.49 ± 1.2 min at 70 °C, 28.9 ± 1.6 min at 80 °C, and 33.8 ± 2.1 min at 90 °C. It was not possible to crystallize this sample at 100 °C. The melting thermograms had two separate peaks, unlike the thermograms of the samples produced on butyric acid, the main peak with a larger area in the zone of higher temperatures, 166 °C, and a small peak at a temperature of 147–150 °C. The crystallization time of the sample synthesized on butyric acid with a similar content of 3HB (10.7 mol.%) was slightly shorter (12.9 ± 1.1 min) at 70 °C; the crystals melted in the temperature range from 141 to 152 °C, and there were two melting peaks on the thermograms, which indicated the presence of two modifications of the crystals. With an increase in temperature to 80 °C, the crystallization time increased to 23.3 ± 1.1 min. At 90 °C, there were no pronounced exothermic effects on the crystallization curves that could be attributed to the formation of the crystalline phase. At the same time, there were weak endothermic effects on the melting thermograms, attributed to melting; it was shown that about 8 ± 1.3% of the sample underwent crystallization. At 100 °C, that sample could not be crystallized.

The crystallization time of the sample synthesized on WFO with a high content of 3HV monomers (22.2 mol.%) was 11.0 ± 2.1 min at 70 °C, 21.0 ± 2.0 min at 80 °C, and 23.1 ±2.1 min at 90 °C. However, no exothermic effect that could be attributed to the crystallization effect was recorded at 100 °C, while the sample had a melting peak. The C_x_ value decreased from 43 ± 2.5% at 70 °C to 11 ± 0.7% at 100 °C. The melting thermogram showed two distinct peaks at 162 °C and 147 °C, i.e., a shift of the peaks to the high-temperature region was noted. The highest melting points were recorded for the crystalline phase obtained at 100 °C, 165 °C, and 155 °C. The crystallization time of the sample synthesized on butyric acid with a 3HV monomer content of 18.9 mol.% was 13.9 ± 1.7 min at 70 °C. The resulting crystalline phase melted in a wide temperature range of 141–166 °C; a wide melting peak with two maxima was recorded on the thermograms. With an increase in the crystallization temperature, there were no pronounced thermal effects on the crystallization thermograms. On the melting thermograms, peaks indicating the presence of a crystalline phase were recorded only for the sample crystallized at 80 °C; the C_x_ value was 3 ± 0.6%. It was not possible to crystallize the sample at 90 °C and 100 °C.

Samples of the copolymer with the highest contents of 3HV monomers (58.8 and 59.7 mol.%), regardless of the type of C-substrate, could not be crystallized under the experimental conditions. Difficulties in isothermal crystallization of mixtures with a high 3HV content were reported by Chan et al. [95]. It was also noted that a melt-quenched sample of P(3HB-*co*-3HV) containing 20 mol.% of 3HV did not crystallize and remained amorphous after 15 min [31].

Experiments performed in the current study showed that the crystallization kinetics of PHA samples with different but close ratios of 3HB and 3HV monomers synthesized on WFO or butyric acid was influenced not only by the content of 3HV monomers, but also by the type of carbon substrate on which the samples were synthesized.

The thermal behavior of the P(3HB-*co*-3HV) samples synthesized on sugars in the culture of the same bacterial strain, *C. necator* B-10646, was similar during exothermic crystallization and depended on the content of 3HV monomers [96]. Under isothermal crystallization conditions, the P(3HB-*co*-3HV) sample with 10 mol.% of 3HV showed low crystallization rates. With increasing crystallization temperature, the crystallization time increased. If at 50 °C the crystallization time was 7.0 min, at 90 °C it was 46.0 min. At 100 °C, the sample remained amorphous for 7 h. Under isothermal crystallization conditions, it was not possible to crystallize samples with a 3HV content of 26.2 mol.% and 48.6 mol.%.

**Figure 9:**
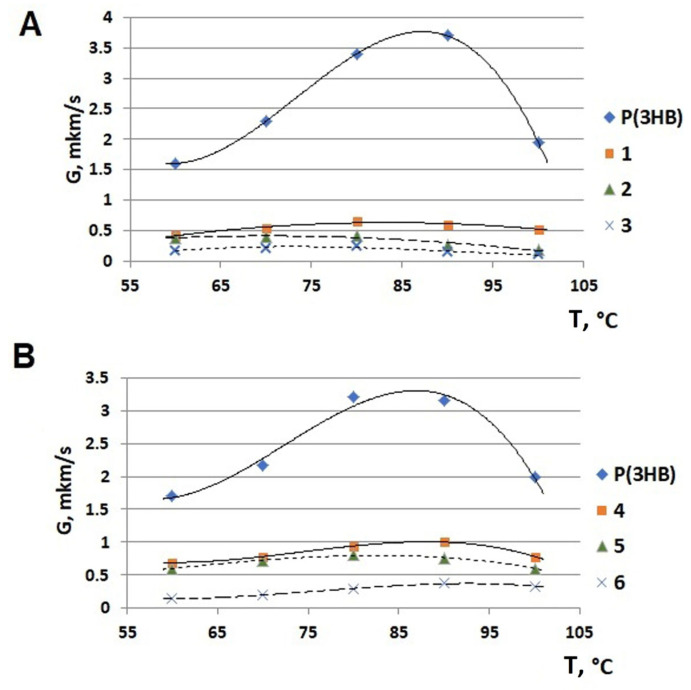
The rate of spherulite formation of P(3HB-*co*-3HV) samples with different contents of 3HB and 3HV monomers synthesized by *C. necator* B-10646 on butyric acid (control) (**A**) and WFO (**B**) (sample numbering according to Table 2).

**Figure 10:**
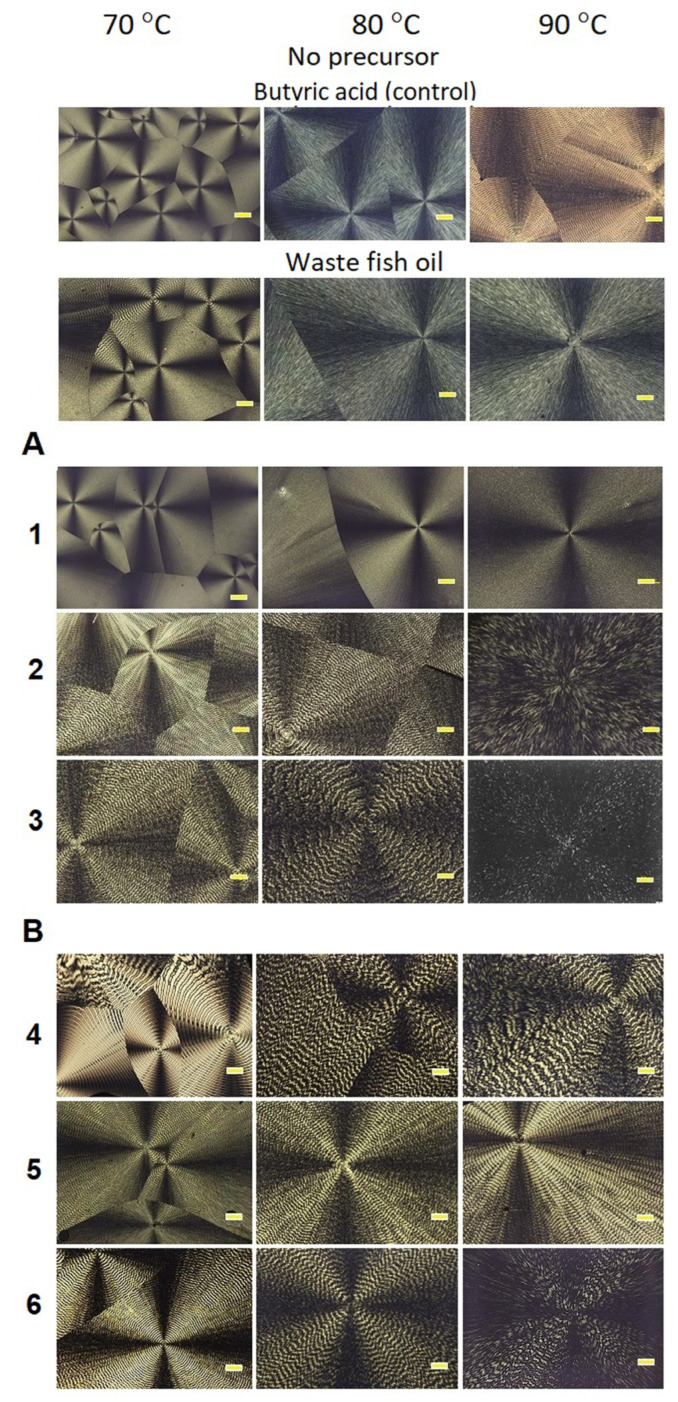
Morphology of spherulites of P(3HB-*co*-3HV) samples with different 3HB and 3HV monomer contents, synthesized by *C. necator* B-10646 on butyric acid (control) (**A**) and WFO (**B**), formed during isothermal crystallization of copolymer melts at different temperatures. Bar 100 μm (sample numbering according to Table 2).

As is known, the most common crystalline form of polymers is spherulites [94], which are formed upon rapid cooling of polymer melts, as well as upon precipitating from concentrated solutions. As PHAs are semicrystalline polymers, their crystallization occurs near the equilibrium state, at which crystalline lamellas develop into spherulites [97]. Polymers of the PHA family are characterized by the formation of extremely large spherulites [98]. The rate of formation, size, and morphology of spherulites are determined by many factors, but the key one is the temperature at which polymer crystallization occurs [99]. The influence of the 3HV monomer content in the P(3HB-*co*-3HV) copolymers synthesized on WFO or butyric acid was shown in the study of the spherulite formation rate and morphology (Figure 9 and Figure 10). A typical picture of the spherulite morphology—“Maltese cross” and concentric stripes—was found in all studied samples of P(3HB-*co*-3HV), regardless of the type of the main carbon substrate used and the ratio of monomers (Figure 10).

The polymer samples synthesized on the two studied C-substrates without precursor additives differed in their chemical composition (Table 2), and that affected the spherulite growth rate. In the three-component sample synthesized on WFO, with a change in crystallization temperature from 60 °C to 100 °C, the spherulite formation rate varied from 1.7 at a temperature of 60 °C to 2.0 μm/s at a temperature of 100 °C, reaching its maximum values (3.22 ± 0.12 μm/s) at a temperature of 85 °C. For the P(3HB) homopolymer sample synthesized on butyric acid, this value varied within the same limits, but the change in the spherulite growth rate with an increase in crystallization temperature occurred much faster and the maximum value of the spherulite formation rate (3.74 ± 0.23 μm/s) could be observed around 85 °C. These differences are likely caused by the fact that in the case of WFO, the synthesized copolymer contained minor inclusions of 3-hydroxyvalerate and 3-hydroxyhexanoate monomers.

The spherulite growth rate in the P(3HB-*co*-3HV) samples synthesized on WFO was highest at a temperature close to 85 °C. At the same time, for the samples with a 3HV monomer content below 50 mol.%, the spherulite growth rate was the highest. That is, with a 3HV content of 11.9 mol.%, the spherulite growth rate was 1.00 ± 0.06 μm/s and at 22.2 mol.% it was slightly lower, 0.78 ± 0.04 μm/s. However, with an increase in the 3HV monomer content to 59.7 mol.%, the maximum value of the spherulite growth rate sharply decreased to 0.35 ± 0.02 μm/s. A similar result was observed in the P(3HB-*co*-3HV) samples synthesized on butyric acid. That is, with a 3HV content of 10.7 and 18.9 mol.%, that value was 0.65 ± 0.03 and 0.42 ± 0.02 μm/s, respectively, and as the 3HV content was increased to 58.8 mol.%, the maximum growth rate of spherulites decreased to 0.27 ± 0.06 μm/s. Thus, in general, the growth rate of spherulites is higher in copolymer samples synthesized on WFO, and this is a positive effect in terms of the quality and processability of the polymer material.

The size of spherulites in the samples synthesized on WFO at 70 °C varied between 300 μm and 700 μm depending on the content of 3HV monomers. At higher crystallization temperatures, larger spherulites were formed, reaching a size of 3.0 mm or greater at 90 °C for the entire studied range of 3HV monomer major inclusions in the copolymer. This is most likely due to the lower nucleation density at higher crystallization temperatures [56]. Similar changes in the morphology of spherulites were also obtained for the samples synthesized on butyric acid. For instance, at a temperature of 70 °C, the size of spherulites varied from 150 to 600 μm in the studied range of 3HV inclusions. At a crystallization temperature of 90 °C and above, the size of spherulites increased to 2.5 mm or larger. In general, the results indicate a less pronounced effect of the type of carbon substrate on the morphology of spherulites compared to the rate of their formation.

As noted above, information on the physicochemical properties of P(3HB-*co*-3HV) copolymers synthesized on WFO is extremely scant. The effects of fatty waste on the thermal behavior of polymers, crystallization patterns, formation and morphology of spherulites have not been previously studied and are not reported in the available literature. This applies not only to the effect of WFO but also other animal- and plant-based fat waste used as a carbon substrate on the properties of PHA. Therefore, a comparison of the obtained results can only be carried out with P(3HB-*co*-3HV) samples synthesized on other C-substrates and other producer strains. For example, the desired characteristics of the P(3HB-*co*-3HV) samples containing 10.0, 26.2, and 48.6 mol.% of 3HV monomers and synthesized in the culture of the same wild-type strain, *C. necator* B-10646, on fructose were comparable: the highest growth rate of spherulites was recorded at 80 °C in the sample with the lowest monomer content. With an increase in the 3HV content, the growth rate of spherulites decreased [96]. A similar relationship was obtained for a series of samples with 3HV monomer content between 12 and 82 mol.% synthesized on complex substrates (sludge hydrolysates or fermented whey permeate) in [56]. Similarly, the highest spherulite growth rate (2.0 μm/s) was recorded for the sample with the lowest 3HV monomer content (8 mol.%) during exothermic crystallization at 80 °C [100]. The data reported in those studies on the relationship between the spherulite growth rate and the 3HV monomer content in P(3HB-*co*-3HV) are consistent with the results of the present work, which demonstrated a decrease in the spherulite growth rate with an increase in the 3HV content in the copolymers synthesized by *C. necator* B-10646 on WFO, as well as on butyric acid.

A series of in-depth studies of the crystallization kinetics and spherulite morphology of P(3HB-*co*-3HV) samples performed by B. Laycock et al. [33,34,35,36,37] showed that this is a complex and multifactorial process, which is influenced not only by the monomer ratio in the copolymer and the crystallization temperature, but also by the patterns of incorporation of 3-hydroxyvalerate monomers into the C-chain of 3-hydroxybutyrate (block or statistically random). This indicates the complexity of the crystallization process of P(3HB-*co*-3HV) copolymers and demonstrates the relevance of the research of this promising representative of the PHA family.

## 4. Conclusions

The present article is a study of the fundamental possibility of synthesizing degradable microbial polymers of the polyhydroxyalkanoate (PHA) family using waste fish oil (WFO) as the main carbon substrate. Synthesis of “green” bioplastics using waste is a contribution to the concept of circular economy and corresponds to modern trends of gradual replacement of synthetic plastics by degradable polymeric materials. For the first time, effective synthesis of P(3HB-*co*-3HV) copolymers was carried out using the wild-type strain *C. necator* B-10646 grown on waste fish oil (WFO) derived from the heads of *Sprattus sprattus balticus* in the production of sprats. Copolymer specimens with significant proportions of 3-hydroxyvaletrate monomers, from 11 to 59%, were synthesized. They had a reduced degree of crystallinity, thermal stability, and molecular weight characteristics typical of P(3HB-*co*-3HV). The present study demonstrated the possibility of productive synthesis of P(3HB-*co*-3HV) copolymers without deterioration of their basic properties using large-tonnage and renewable waste from the fish canning industry as the main carbon substrate, which is a way to expand the raw material supply, reduce costs for producing promising bioplastics, and expand the areas of their application.

## Figures and Tables

**Table 1:** P(3HB-*co*-3HV) synthesis parameters of *C. necator* B-10646 cells grown on butyric acid (control) and WFO with the addition of potassium valerate as a precursor of 3HV monomers.

Number of Potassium Valerate Additions, ⅀ Concentration (g·L^−1^)	X (g·L^−1^)	P(3HB-*co*-3HV)		3HV (mol.%)	Productivity (g·L^−1^·h^−1^)	
		(g·L^−1^)	(%)		Biomass, P_X_	P(3HB-*co*-3HV), P_P(3HB/3HV)_
Butyric acid (control)						
No precursor	4.3 ± 0.2	3.1 ± 0.2	72 ± 3	-	0.090 ± 0.004	0.065 ± 0.004
1 addition (0.5 g·L^−1^), total 0.5 g·L^−1^	4.5 ± 0.2	3.2 ± 0.2	70 ± 3	10.7 ± 0.4	0.094 ± 0.004	0.066 ± 0.004
2 additions (0.7 + 0.7 g·L^−1^), total 1.4 g·L^−1^	4.6 ± 0.2	3.3 ± 0.3	71 ± 4	18.9 ± 0.4	0.096 ± 0.004	0.069 ± 0.006
3 additions (1.0 + 1.0 + 2.0 g·L^−1^), total 4.0 g·L^−1^	3.6 ± 0.1	2.4 ± 0.2	67 ± 3	58.8 ± 2.1	0.074 ± 0.002	0.050 ± 0.004
Waste fish oil						
No precursor	4.2 ± 0.3	2.7 ± 0.2	66 ± 3	1.1 ± 0.1	0.087 ± 0.005	0.057 ± 0.005
1 addition (0.5 g·L^−1^), total 0.5 g·L^−1^	3.1 ± 0.3	1.9 ± 0.1	61 ± 2	11.9 ± 0.4	0.065 ± 0.006	0.039 ± 0.003
2 additions (0.7 + 0.7 g·L^−1^), total 1.4 g·L^−1^	3.3 ± 0.3	2.0 ± 0.2	63 ± 2	22.2 ± 1.5	0.068 ± 0.007	0.042 ± 0.004
3 additions (1.0 + 1.0 + 2.0 g·L^−1^), total 4.0 g·L^−1^	2.2 ± 0.3	1.1 ± 0.2	49 ± 2	59.7 ± 3.1	0.047 ± 0.005	0.023 ± 0.003

**Table 2:** Chemical composition and properties of PHA as dependent on the ratio of monomers in polymer synthesized by bacteria *C. necator* B-10646 on various carbon sources.

No.	Potassium Valerate (g·L^−1^)	PHA Composition, mol.%			M_n_, kDa	M_w_, kDa	Đ	C_x_, %	T_melt_, °C	T_cryst_, °C	T_g_, °C	T_degr_, °C
		3HB	3HV	3HHx								
Without precursors												
Butyric acid		100	0	0	190 ± 6	418 ± 8	2.2	62.0 ± 2.0	173.2 ± 0.2	70.6 ± 2.2	– ^1^	281.9 ± 2.3
WFO		98.6 ± 0.2	1.0 ± 0.1	0.4 ± 0.0	219 ± 8	528 ± 7	2.4	45.1 ± 1.0	168.7 ± 0.1	63.6 ± 1.1	4.0 ± 0.5	282.7 ± 2.2
Butyric acid												
1	0.5	89.3 ± 0.5	10.7 ± 0.4	0	194 ± 16	458 ± 6	2.4	50.4 ± 2.2	140.7 ± 0.3 151.5 ± 0.2	67.2 ± 1.6	0.4 ± 0.1	142.4 ± 1.5 280.4 ± 3.1
2	1.4	81.1 ± 0.4	18.9 ± 0.4	0	165 ± 9	430 ± 5	2.6	30.2 ± 2.7	141.1 ± 0.2 166.4 ± 0.3	78.5 ± 2.1	−1.1 ± 0.2	119.6 ± 1.3 268.3 ± 2.5
3	4.0	41.2 ± 1.7	58.8 ± 2.1	0	193 ± 10	520 ± 9	2.7	–	–	–	−16.6 ± 0.4	128.1 ± 1.3 271.8 ± 2.1
WFO												
4	0.5	88.1 ± 0.7	11.9 ± 0.4	0	130 ± 5	390 ± 1	3.0	32.2 ± 1.2	165.8 ± 0.2	61.2 ± 2.9	−1.4 ± 60.1	280.7 ± 2.1
5	1.4	77.8 ± 1.7	22.2 ± 1.5	0	206 ± 2	573 ± 9	2.6	36.5 ± 3.5	156.0 ± 0.3	53.6 ± 2.3	−3.9 ± 60.2	275.2 ± 2.8
6	4.0	40.3 ± 2.2	59.7 ± 3.1	0	142 ± 10	417 ± 8	2.9	3.5 ± 0.9	163.9 ± 0.1	–	−9.0 ± 0.1	270.4 ± 1.7

^1^ Not determined.

## Data Availability

All data are available in the paper.

## Supplementary Materials

The following supporting information can be downloaded at: https://www.mdpi.com/article/10.3390/polym17162171/s1, Table S1: Composition of fatty acids in waste fish oil obtained from processing Baltic sprat (*Sprattus sprattus balticus*) using enzymatic method (% of the sum of FAs); Table S2: Two-way analysis of variance of the results of the effect of carbon substrate (WFO and butyric acid) and the concentration of the precursor ɛ-caprolactone on the production parameters of the bacterial culture *C. necator* B-10646.
