# Supplementary material for: Synthesis and Properties of Degradable Poly(3-hydroxybutyrate-co-3-hydroxyvalerate) [P(3HB-co-3HV)] Derived from Waste Fish Oil
Source: Polymers (Basel). 2025 Aug 8;17(16):2171. doi: 10.3390/polym17162171 (PMC12389658; doi:10.3390/polym17162171)
Supplement: Supplementary file 1 [file polymers-17-02171-s001.zip › Table S1.pdf]

Article

Synthesis and Properties of Degradable Poly(3-hydroxybutyrate-*co*-3-hydroxyvalerate) [P(3HB-*co*-3HV)] Derived from Waste Fish Oil

Tatiana G. Volova [1,2], Evgeniy G. Kiselev [1,2], Alexey G. Sukovatyi [1,2], Natalia O. Zhila [1,2,*], Kristina Yu. Sapozhnikova [1,2], Natalia D. Ipatova [1,2] and Peter O. Shishatskii [1]

[1] Institute of Biophysics SB RAS, Federal Research Center "Krasnoyarsk Science Center SB RAS", 50/50 Akademgorodok, Krasnoyarsk 660036, Russia; volova45@mail.ru (T.G.V.); evgeniygek@gmail.com (E.G.K.); a.sukovatiy@yandex.ru (A.G.S.); kristina.sap@list.ru (K.Y.S.); ipatovahatal@gmail.com (N.D.I.);shishatskaya@inbox.ru (P.O.S.)
[2] Basic Department of Biotechnology, School of Fundamental Biology and Biotechnology, Siberian Federal University, 79 Svobodnyi Av., Krasnoyarsk 660041, Russia
* Correspondence: nzhila@mail.ru; Tel.: +7-391-290-54-91; Fax: +7-391-243-34-00

Table S1. Composition of fatty acids in waste fish oil obtained from processing Baltic sprat (*Sprattus sprattus balticus*) using enzymatic method (% of the sum of FAs).

Fatty acid	Content of fatty acid
14:0	4.35
16:0	25.05
16:1ω7	0.30
17:0	0.29
17:1	0.36
18:0	1.48
18:1ω9	28.09
18:1ω7	2.28
18:2ω6	4.95
18:3ω3	2.82
20:0	0.32
20:1ω9	1.19
20:2	0.39
20:3	0.22
20:4	0.69
20:5ω3	9.08
22:0	0.37
22:1	0.41
22:6ω3	13.71
24:1ω9	2.24
Others[1]	1.41
∑saturated FAs	33.27
∑unsaturated FAs	66.73
∑saturated FAs/∑unsaturated FAs	0.50

[1] i-14:0, ai-14:0, ai-16:0 FAs.
