# Supplementary material for: Synthesis and Properties of Degradable Poly(3-hydroxybutyrate-co-3-hydroxyvalerate) [P(3HB-co-3HV)] Derived from Waste Fish Oil
Source: Polymers (Basel). 2025 Aug 8;17(16):2171. doi: 10.3390/polym17162171 (PMC12389658; doi:10.3390/polym17162171)
Supplement: Supplementary file 1 [file polymers-17-02171-s001.zip › Table S2.pdf]

Article

Synthesis and Properties of Degradable Poly(3-hydroxybutyrate-*co*-3-hydroxyvalerate) [P(3HB-*co*-3HV)] Derived from Waste Fish Oil

Tatiana G. Volova [1,2], Evgeniy G. Kiselev [1,2], Alexey G. Sukovatyi [1,2], Natalia O. Zhila [1,2,*], Kristina Yu. Sapozhnikova [1,2], Natalia D. Ipatova [1,2] and Peter O. Shishatskii [1]

[1] Institute of Biophysics SB RAS, Federal Research Center "Krasnoyarsk Science Center SB RAS", 50/50 Akademgorodok, Krasnoyarsk 660036, Russia; volova45@mail.ru (T.G.V.); evgeniygek@gmail.com (E.G.K.); a.sukovatiy@yandex.ru (A.G.S.); kristina.sap@list.ru (K.Y.S.); ipatovahatal@gmail.com (N.D.I.);shishatskaya@inbox.ru (P.O.S.)

[2] Basic Department of Biotechnology, School of Fundamental Biology and Biotechnology, Siberian Federal University, 79 Svobodnyi Av., Krasnoyarsk 660041, Russia

* Correspondence: nzhila@mail.ru; Tel.: +7-391-290-54-91; Fax: +7-391-243-34-00

Table S2. Two-way analysis of variance of the results of the effect of carbon substrate (WFO and butyric acid) and the concentration of the precursor (potassium valerate) on the production parameters of the bacterial culture *C. necator* B-10646.

Source of variation	SS	df	MS	F	P-Value	F critical
			**X, g·L^{-1}**			
Substrate	6.72	1	6.72	129.03	4.54E-9	4.49
Precursor concentration	6.03	3	2.01	38.6	1.49E-79	3.24
Interaction	1.59	3	0.53	10.20	5.36E-4	3.24
			**P(3HB-*co*-3HV), %**			
Substrate	728.20	1	728.20	116.53	9.38E-09	4.49
Precursor concentration	357.76	3	119.25	19.08	1.55E-0.5	3.24
Interaction	158.81	3	52.94	8.47	0.001	3.24
			**3HV, mol.%.**			
Substrate	14.88	1	14.88	7.01	0.02	4.49
Precursor concentration	11772.66	3	3924.22	1848.14	1.57E-20	3.24
Interaction	6.01	3	2.00	0.94	0.44	3.24
